# Bioactives and Traditional Herbal Medicine for the Treatment of Cardiovascular/Cerebrovascular Diseases 2015

**DOI:** 10.1155/2015/320545

**Published:** 2015-08-26

**Authors:** Joen-Rong Sheu, Pitchairaj Geraldine, Mao-Hsiung Yen

**Affiliations:** ^1^Graduate Institute of Medical Sciences, College of Medicine, Taipei Medical University, Taipei 110, Taiwan; ^2^Department of Animal Science, Bharathidasan University, Tiruchirappalli, Tamil Nadu 620 024, India; ^3^Department of Pharmacology, National Defense Medical Center, Taipei, Taiwan

Cardiovascular diseases (CVDs) are still the principal cause of death worldwide. Weakened endothelial function followed by inflammation of the vessel wall hints at atherosclerotic lesion formation that causes myocardial infarction and stroke. Heart failure can arise as consequence of large myocardial infarctions. In its more severe stages, heart failure patients have a life anticipation that is parallel to destructive cancers. Accordingly, the increase in risk factor load by metabolic diseases and age augments the incidence for vascular and cardiac diseases and provides a challenge for developing efficient treatments. There is widespread proof to show that drug treatment of conventional risk factors is effective in reducing cardiovascular events. More effective treatment of CVD with various classes of antihypertensive drugs has been associated with greater benefits, but some recent studies suggest we may be reaching the optimal level of treated blood pressure in some patient groups. Apart from the treatment of cardiovascular risk factors with pharmacological agents and the use of antithrombotic drugs, there is growing awareness of the role of dietary factors and herbal medicines in the prevention of CVD and the possibility of their use in treatment. Investigators from different places of the world like China, Taiwan, Bangladesh, Pakistan, and so forth contributed to this special issue by presenting tremendous papers. These papers deliver an analysis in this field and create innovative contributions concerning the mechanism of action of bioactives and traditional herbal medicine for the treatment of cardiovascular/cerebrovascular diseases.

Some interesting papers in this special issue address the cardioprotective effects of Chinese herbal medicine and natural compounds. For instance, a paper summarized the synergetic cardioprotective potential of herbal combination of four plants, namely,* Terminalia arjuna*,* Cactus grandiflorous*,* Crataegus oxyacantha*, and* Piper nigrum* through curative and preventive mode of treatment analysis and this paper reported preadministration and postadministration of herbal mixture restore the levels of biomarker of cardiotoxicity, which includes cardiac marker enzymes, lipids profile, and antioxidant enzymes. Similarly, another paper in this issue reports the cardioprotective effects of Sundarban honey on cardiac troponin I, cardiac marker enzymes, the lipid profile, lipid peroxidation products, and histoarchitecture of the myocardium against isoproterenol-induced myocardial infarction in Wistar rats. Pinggan Qianyang recipe (PQR), a Chinese medicine recipe, has long been used for calming the liver. It has also been used to treat essential hypertension with satisfactory results. Consistent with this concern, this special issue published a paper that reports PQR exerts its antihypertensive effect through deterioration of the vascular remodeling process. The mechanism might be associated with regulating differentially expressed miRNAs in aorta tissue.

Despite the fact that there are major developments in treating ischemic stroke over the last decade, stroke is still a serious concern for which effective drug therapy is not yet available. In the search for neuroprotective agents from natural sources, a number of plant extracts and several natural products were isolated and reported to provide neuroprotection against ischemic stroke. A few papers in this special issue report the neuroprotective effects of Chinese herbal medicine and natural compounds. For instance,* Antrodia camphorata* (*A. camphorata*), a fungus generally used in Chinese folk medicine for the treatment of viral hepatitis and cancer, has shown neuroprotective effects in embolic rats. This effect may correlate with the downregulation of the iNOS, HO-1, Bax, and activated caspase-3 and the inhibition of OH° signals. Another study shows alpha-lipoic acid attenuates middle cerebral artery occlusion-induced cerebral ischemia and reperfusion injury via insulin receptor-dependent and PI3K/Akt-dependent inhibition of NADPH oxidase. Moreover, an interesting study in this special issue established the effects of tetramethylpyrazine (TMP) on functional recovery and neuronal dendritic plasticity after experimental stroke. In this study, the authors have shown that enhanced dendritic plasticity contributes to TMP-elicited functional recovery after ischemic stroke.

Hinokitiol is a naturally occurring compound isolated from the wood of* Chamaecyparis taiwanensis*. It is involved in multiple biological activities, including antimicrobial and antitumorigenic activities. Although hinokitiol has been reported to inhibit inflammation, its immunological regulation in lymphocytes remains inadequate. With this context, a well-designed study reported that hinokitiol downregulated cyclin D3, E2F1, and Cdk4 expression and upregulated p21 expression in concanavalin A- (ConA-) stimulated T lymphocytes. It further demonstrated that hinokitiol upregulates p21 expression and attenuates IFN-*γ* secretion in T lymphocytes from the spleens of mice, thereby arresting the cell cycle in the G0/G1 phase. These authors concluded that hinokitiol provides benefits in treating patients with autoimmune diseases. We expect that this special issue grants inventive awareness to increase the therapeutic value of herbal and/or Chinese medicines for treatment or prevention of cardiovascular and ischemia-reperfusion injury-related disorders.

*Joen-Rong Sheu* *Joen-Rong Sheu* *Pitchairaj Geraldine* *Pitchairaj Geraldine* *Mao-Hsiung Yen* *Mao-Hsiung Yen*

